# Developmental Profiles of Mucosal Immunity in Pre-school Children

**DOI:** 10.1155/2010/196785

**Published:** 2010-12-27

**Authors:** Patricia Ewing, Diana C. Otczyk, Stefano Occhipinti, Jennelle M. Kyd, Maree Gleeson, Allan W. Cripps

**Affiliations:** ^1^Faculty of Health, University of Canberra, ACT, 2601, Australia; ^2^Griffith Health Institute, School of Medicine, Griffith Health, Griffith University, Gold Coast, QLD, 4222, Australia; ^3^Griffith Health Institute, School of Psychology, Griffith Health, Griffith University, Gold Coast, QLD, 4222, Australia; ^4^Capricornia Centre for Mucosal Immunology, Central Queensland University, Rockhampton, QLD, 4702, Australia; ^5^Hunter Institute Medical Research, University of Newcastle, Newcastle, NSW, 2308, Australia

## Abstract

This study investigated the effect of attending pre-school on mucosal immunity. Children 3.5 to 5 years of age who attended pre-school were observed for a 10 month period. Demographic information was collected on previous childcare experiences, the home environment and clinical information relating to the child and the family. A daily illness log was kept for each child. A multivariate longitudinal analysis of the relation between immunoglobulins in saliva and age, gender, childcare experience, pre-school exposure, number of siblings, environmental tobacco smoke (ETS), atopy and hospitalisation was conducted. There was a positive association of higher IgA levels with the winter season and with children being older than 4 years (*P* < .001), having attended childcare prior to commencing pre-school (*P* < .05), and having been exposed to ETS at home (*P* < .05). Lower IgA levels were associated with being atopic (*P* < .05). Higher IgG levels were associated with exposure to ETS (*P* < .001), while lower levels were associated to having atopy. Higher IgM levels were associated with previous childcare experience (*P* < .01) whilst having been hospitalised was associated with having low salivary IgM levels (*P* < .01). Lagged analyses demonstrated that immunological parameters were affected by the number of respiratory infections in the preceding 2 months.

## 1. Introduction

The mucosal immune system begins to develop shortly after conception, and whilst structurally complete at birth, it is functionally immature [1]. Functional development is rapidly stimulated after birth by the ingestion and inhalation of bacterial and food antigens and mitogens. The salivary glands have long been recognised as part of the common mucosal immune system [2], and as such, salivary secretions have been often used to assess mucosal immune competence in humans. Numerous studies have looked at the development of the mucosal immune system in the early years of childhood in order to gain some insight into the maturation of the host mucosal defences in children. Whilst some have shown that salivary immunoglobulin levels continue to increase throughout the childhood years [3], others have found that adult levels are reached by seven years [4–8] and even as early as 12 to 18 months of age [9, 10]. Although the literature is not conclusive on the postnatal ontogeny, what is clear is that the timing and pattern of the increase from infant to adult levels appear to depend on environmental influences [11–14]. 

The development of mucosal immunity is profoundly affected by exposure to infection. The ontogeny patterns for mucosal antibodies in young children appear to reflect the degree of antigenic exposure in their environment. Increased antigenic exposure of infants through poor hygiene and health in developing countries [13, 14] and hospitalisation or daycare attendance in developed countries [9] results in high levels of antibodies in saliva that can occur at an earlier age [13, 14]. Early life events appear to have a critical influence on the ultimate pattern of immune maturation [1], and there has been a pressing need for longitudinal studies to understand how immune responses evolve in children and the impact on long-term clinical outcomes. However, to date, much of the information regarding the impact of environmental stimuli on the developing immune system has been anecdotal. The common interpretation is that older siblings and childcare attendance are indirect measures of early life infections and that early life infections have a preventative effect on the development of atopic disorders [15] and infectious illness. This study was designed to examine several factors that may impact the development of mucosal immunocompetence in young children. Children were studied during their pre-school year as this would be their first encounter with the increased antigenic exposure associated with starting school. Children aged 3.5 to 5 years attending pre-school were monitored for one school year (February to December) to ascertain whether any alteration in mucosal immune function could be linked to environmental, developmental, and clinical events. The effects were monitored using various markers of potential mucosal immune stimuli, such as the number of siblings in the family, days of attendance at daycare facilities, and known modifiers of mucosal immunity such as environmental tobacco smoke (ETS) exposure. A record was kept of the general health status of the children during the year and episodes of gastrointestinal tract (GIT), and respiratory (RT) symptoms were recorded by the parents. Saliva samples were collected at regular intervals throughout the year and were analysed for immunoglobulin levels IgA, IgG, and IgM and *Escherichia coli*-specific IgA antibodies.

## 2. Methods

### 2.1. Study Population

After obtaining written informed consent, 37 children, aged 3.5–5 years, were recruited from a single pre-school on the south coast of NSW, Australia. All of the children had been fully immunised in accordance with the Australian National Health and Medical Research Council recommended vaccination schedule [16]. The pre-school had a total annual enrolment of 100 to 120 children, aged between 3 and 6 years of age. Class sizes were approximately 25 children each day. Ethical approval for the study was provided by the Committee for Ethics in Human Research at the University of Canberra.

### 2.2. Data Collection

Information on the child's health and environmental exposures was collected from parents by questionnaires prior to commencement of the study. The baseline questionnaires collected three sets of information: individual, family, and clinically related information. Individual characteristics included child's age, gender, number of days the child would be attending pre-school, previous pre-school or childcare centre arrangements. Previous childcare arrangement was defined as a formal childcare centre. Family characteristics included number and age of siblings, and number of people who smoked tobacco in the home. Clinically related information included: atopy defined as eczema, asthma, urticaria, or known allergy to food or inhaled allergens; medications taken by the child; previous major illness, hospitalization, or dental treatment.

The child's age was measured in months and was grouped as a 2-category classification: children that were 4 years and younger and children that were older than 4 years.

### 2.3. Infection Data

Saliva samples were collected in February, April, July, September, and December, and collections were spread over three days of the week on each occasion. The pattern of attendance at the pre-school ensured that the same children were always present on Day 1, Day 2, and Day 3, respectively, so the number of days between saliva collection events was the same for all children. Infection records were related to saliva samples by dividing the year into four periods, these being the periods between one saliva collection event and the next. Infection records started from the beginning of February when the first saliva samples were collected. This was the beginning of the school year, in the latter part of the Australian summer season.

### 2.4. Infectious Exposures

#### 2.4.1. Respiratory and Gastrointestinal Symptoms

Over the 10-month study period from February to December, parents were asked to record any symptoms of RT or GIT infections on a calendar for each month. An infectious episode was defined as the acute occurrence of a new symptom lasting for at least 1 day with fever. A visit to the doctor and temperature was recorded if the child was ill. GIT illness-related symptoms included: fever, vomiting, diarrhoea and tummy ache. RT symptoms included: fever, vomiting, runny nose, cough, wheezing or whistling breathing, difficulty breathing, and ear ache.

#### 2.4.2. Indirect Measures of Exposure to Infectious Agents

Indirect measures of exposure to infectious agents included: previous attendance at a childcare centre (measured as the number of days per week at a childcare centre and categorized as “low”—<2 days per week for <1 year or “high”—≥2 days per week for ≥1 year); number of days in attendance at pre-school (measured as 1 or 2 days per week); any history of hospitalisation or intrusive medical procedures; older siblings (measured as birth order when the child was included in the study cohort), and number of siblings in the family. The categorization of these measures of exposure is presented in Table 1.

### 2.5. Covariates

The determinants of interest included gender, atopy, and ETS exposure. Atopy was defined as a history of eczema, asthma, urticaria, and allergy. Exposure to ETS was defined as living in the same household as ≥1 smoker. The categorization of these covariates is presented in Table 1.

### 2.6. Laboratory Analyses

#### 2.6.1. Sample Collection

Saliva samples were collected as previously described in [17] on 5 occasions during the school year in the months of February, April, July, September, and December. All samples were collected between 0900 and 1100 hours according to standardised procedures [18, 19]. Approximately 1 mL of saliva was collected from each child on each occasion and stored at −70°C for later analysis of the immunoglobulins IgA, IgG, IgM and *E. coli*-specific IgA antibody levels and total protein concentration.

#### 2.6.2. Measurement of IgA, IgG, and IgM in Saliva

IgA, IgG and IgM were measured in saliva by an in-house indirect enzyme-linked immunosorbent assay (ELISA) as previously described in [17]. 

#### 2.6.3. Measurement of IgA Specific Antibodies to E. coli O Antigen in Saliva

An in-house ELISA measured IgA antibodies against *E. coli* O antigens as described previously [7]. 

#### 2.6.4. Measurement of Total Salivary Protein

The commercially available BCA protein assay kit (Pierce Biotechnology Inc, Rockford, Illinois, USA) was used to measure the total protein concentration of the saliva samples.

### 2.7. Statistical Analysis

Hypotheses were tested with a series of mixed effects multilevel regressions performed using the *xtmixed* procedure of Stata [20]. Multilevel modelling is a contemporary approach to longitudinal designs [21] such as that used in the present study that provides flexibility in analysing hierarchical data sets. The use of the multilevel approach maximises available data in that protocols from any child whose provided readings are included in the estimation.

A separate analysis was conducted for each of the log-transformed immunoglobulin outcomes as well as for total number of illness events (untransformed). In each analysis, a time variable representing the five sample points coded as 1 to 5 was entered into the model to represent linear change in the outcome over the year. As well, the square of the time variable was entered to account for curvilinear change. The covariates were entered, and where necessary, were coded as representative variables for age (≤4 years of age versus >4 years of age); gender (male versus female); having attended previous childcare (no versus yes); amount of pre-school exposure (“low”—<2 days per week for <1 year versus “high”—≥2 days per week for ≥1 year); siblings (no versus yes); parental smoking (no versus yes); atopic (no versus yes); previous hospitalisation (no versus yes). These predictors accounted for differences in levels of the salivary immunoglobulin outcomes over the year. The corresponding multilevel regression coefficients were calculated and are shown in Table 3. In order to examine further effects of the predictors on the rate of change in immunological outcomes over the course of the year, a set of products were calculated from the time variable, and each of the respective predictors and these products were entered as predictors in their own right. Predictors were not entered prior to the formation of the products as most were representative variables (see above) or otherwise contained meaningful zero points. Any significant changes observed in the analyses are reported in results. The effects of mucosal illness on immunoglobulin levels were examined using a series of lagged analyses in which the number of prior RT and GIT infections reported at each sampling point was entered in multilevel mixed-effects regressions with a lag of approximately 2 months to each immunoglobulin outcome.

To allow for the dilution effect of differing flow rates, total salivary immunoglobulin levels were analysed both corrected and uncorrected with regard to total protein levels. No difference was found in the results obtained for the corrected and uncorrected data (data not shown). The salivary immunoglobulin data presented for this study is uncorrected for total protein levels (Table 2).

## 3. Results

### 3.1. Demographics of the Study Cohort

The general characteristics for the children in the study are provided in Table 1. Of the total 37 children, 46% of children were 48 months of age or younger, and 55% were older than 48 months but no older than 60 months. There were approximately equal numbers for each gender, 57% females and 43% males. The majority of children (78%) had previously attended formal childcare prior to starting pre-school. About two thirds of the children studied attended pre-school for 2 days per week, and about one third were attending 1 day per week. More than half of the children had siblings, and over a third were reported to live in a household with a smoker. Approximately 16% of children had an allergic disease, and 38% had previously been hospitalised.

### 3.2. Seasonal and Age-Related Changes in Salivary Immune Parameters

A total of 164 saliva samples, were collected for statistical [22] analysis. Twenty-two children (59.5%) had a complete set of 5 saliva samples and a further 6 (16.2%) had 4 saliva samples collected. Table 2 shows the geometric mean of salivary IgA, IgG, and IgM and *E. coli*-specific IgA at each of the 5 assessment points during the school year in the months of February, April, July, September, and December with the July assessment point representing the southern hemisphere winter. The salivary immunoglobulin concentrations were compared to age-related reference ranges. The salivary IgA levels were predominantly within the reference range, with levels above the population age reference range being recorded for 20 (11.5%) samples. The highest level recorded was 80.4 mg/L. Ten of the samples with elevated levels of total IgA were collected in July. There was a strong seasonal rise in salivary IgA associated with the southern hemisphere winter (Table 2). Significant linear (regression coefficient 0.172 (standard error 0.071; *P* < .05) and curvilinear (regression coefficient −0.043 (standard error 0.071; *P* < .05) changes were found for the total salivary IgA levels only (Table 3). The concentration of salivary IgA remained relatively stable during the time period from February to April (geometric mean 21.83 mg/L; 95% confidence interval (CI), 18.99–25.10 and 21.07 mg/L; 95% CI, 18.20–24.40, resp.), then increased to a mean high of 30.27 mg/L (95% CI, 25.72–35.61) during July, and then showed a downward trend to 22.12 mg/L (95% CI 18.85–25.97) in December, similar to those values seen at the beginning of the pre-school year.

The levels of salivary IgG and IgM measured in this study were all within their respective age-related reference ranges. There was no association between salivary IgG or IgM levels and the time of year that the samples were collected. The IgG level in saliva showed a nonsignificant downward trend from 2.99 mg/L (95% CI, 2.34–3.82) at the start of the school year to a low of 2.32 mg/L (95% CI, 1.86–2.91) during July followed by a high of 3.03 mg/L (95% CI, 2.26–4.06) in September, after which time it returned in December to levels observed in February. Salivary IgM levels tended to fluctuate during the course of the year from 0.87 mg/L (95% CI, 0.64–1.17) in February to 1.10 mg/L (95% CI, 0.81–1.48) in December, but showed no significant trend over the year.

Levels of *E. coli*-specific IgA antibodies were measured using antigen pooled from four enteric strains of the bacterium. No reference range has been established for this assay. Saliva levels of *E. coli*-specific IgA antibodies measured in pre-school children tended to remain relatively stable during the course of the pre-school year from a low geometric mean of 0.08 EU/mL to a high of 0.12 EU/mL.

### 3.3. Multivariate Longitudinal Analysis

The results of the multivariate longitudinal analysis of the relationship between salivary IgA, IgG, and IgM and *E. coli*-specific IgA antibodies, and age, gender, prior childcare experience, length of pre-school attendance, number of siblings in the family, ETS exposure, atopy, and hospitalisation are provided in Table 3. Longitudinal analysis confirmed the strong positive association of higher IgA levels, but not the trajectory of these over time, with children being aged older than 4 years at the onset of the study (*P* < .001), having attended childcare prior to commencing pre-school (*P* < .05), and having been exposed to ETS at home (*P* < .05). Lower salivary IgA levels were strongly associated with being atopic (*P* < .05). There was no significant association between the salivary IgA levels and gender, length of pre-school attendance, number of siblings in the family, or having been hospitalised. There was a significant association between higher salivary IgG levels and having been exposed to ETS (*P* < .001). Lower salivary IgG levels were associated with higher number of days in attendance at pre-school (*P* < .05) and having atopy (*P* < .05). Higher salivary IgM levels were associated with previous childcare experience (*P* < .01). A history of having been hospitalised was associated with having low salivary IgM levels (*P* < .05). Higher *E. coli*-specific IgA levels were associated with previous childcare experience (*P* < .05) and higher attendance days at pre-school (*P* < .01). Female gender was correlated to having lower *E. coli* antibody levels. None of the rates of change of immunological variables were associated with any of the covariates examined in the study.

### 3.4. Effect of Infection

Records of illness in the children were studied for possible correlation with salivary antibody levels. Compliance varied greatly with 23 (56.1%) of the children having a complete record for the whole 10 months of the study. Of the children who were included in the statistical analysis, 62.2% had complete infection data records for the study period. In the study population, 34 of the children (91.9%) experienced at least 1 RT symptom during the school year. As expected, during the winter flu season, children were more likely to experience an RT illness with a total of 39.3% of episodes occurring during the months of May, June, and July. Twenty-eight (75.7%) children experienced a RT infection during the winter months. The incidence of RT infections reported was highest in the month of June (23.4%). Thirty-one (83.8%) children experienced at least one GIT symptom during the school year. The majority of episodes occurred in the early part of the year when the children were newly enrolled in pre-school. The number of episodes fell from a high of 20.0% of children with reported cases in February to a low of 4.4% in October. Approximately one third of the children (35.1%) experienced a GIT infectious episode with 35.0% of infections occurring in the first month of pre-school. Lagged analyses showed that immunoglobulin levels were primarily affected by the number of RT infections in the preceding two months, and not, by GIT infections. There was a strong inverse relationship between the number of RT infections and the levels of salivary IgA and IgG. The corresponding regression parameters (and standard errors) for RT infections for salivary IgA and IgG, respectively, were −0.044 (standard error 0.021; *P* = .039) and −0.085 (standard error 0.031; *P* = .007).

## 4. Discussion

This study of 37 children aged 3.5–5 years attending a single Australian pre-school for one school year establishes longitudinal data on (1) the development of total salivary immunoglobulins IgA, IgG, and IgM, and IgA antibody specific against *E. coli* O antigen; (2) the influence of age on these parameters; (3) the impact of season and infection on immune parameters; (4) environmental factors which affect the pattern of immunoglobulin ontogeny and antibody production.

Using a standardised protocol [18], saliva samples were collected from pre-school children at 5 different time intervals during the school year. Immunoglobulin levels were found not to differ significantly at the end of the study compared to those at the beginning of the year. The geometric mean levels for IgA, IgG, and IgM during the course of the study varied between 20–30 mg/L, 2.3–3.0 mg/L, and 0.8–1.1 mg/L, respectively, similar to those previously reported for children in the same age range [22]. Children older than 4 years of age had higher levels of salivary IgA when compared with younger children. This observation is consistent with previous studies of mucosal immune ontogeny [7, 9, 14, 23]. The lack of increase in immunoglobulin levels during the course of the pre-school year is in contrast with the findings of a larger cross-sectional study of Australian children conducted in the early 1980s [7] which showed a dramatic increase in salivary IgA levels in children who completed their first year of school. It is speculated that the cohort of children studied previously may have been immunologically naïve and showed a marked increase in mucosal immunoglobulin levels as a result of a large increase in antigenic exposure associated with starting school. However, social and education patterns have changed since this earlier study, and many children now spend some time in childcare centres from an early age with the majority of Australian children spending at least a year in pre-school before they start their formal school education. It would seem that children today are exposed to a wide range of environmental antigenic challenges at an earlier age. 

To assess the pattern of development of specific immunity in the mucosal system to a common gut antigen, IgA antibodies specific to *E. coli* somatic O antigen were measured in saliva. Consistent with earlier studies [7, 9], low levels of *E. coli-*specific IgA O antigen antibodies were detected in the saliva of the children in this study. These levels remained relatively constant during the course of the pre-school year suggesting universal exposure to this bacteria and a closely controlled mucosal response to a major colonizing antigen.

Similar to other studies of pre-school-aged children [24–27], respiratory and enteric illnesses were common in this study group. Increased exposure to other children, and to the common pathogens they carry, has been identified as the primary risk factor for elevated rates of illness among children in group care [24, 26–29], in particular, GIT illness [27–29], RT, and ear infections [24, 27, 29, 30]. There is some anecdotal evidence to suggest that immunity acquired in these settings might protect the child on entrance to primary school [26]. In this study, longitudinal observation of children's symptoms showed more bouts of RT illness in the winter months which was significantly associated with elevated salivary IgA levels. Levels of salivary immunoglobulins are known to change rapidly during the course of an infection, particularly with respect to respiratory infections [18, 31–33]. Total salivary IgA levels have been shown to increase within 1–4 days of the onset of upper respiratory tract symptoms and to fall to preinfection levels within 12 days of appearance of symptoms [18]. This response to infection tends to be large and polyclonal when the antigenic challenge is new. Lagged analysis of the longitudinal data showed that salivary IgA and IgG levels were primarily affected by the number of respiratory infections in the preceding 2 months. Those children with low levels of salivary IgA and IgG had the greater number of RT infections. Similar observations have been made in high performing athletes [34] and in children prone to respiratory infections [35]. More than a third of GIT infections were reported in the first month of the pre-school year. This may reflect exposure to microbial flora not previously encountered and corresponds to previous findings that the pathogens associated with GIT illness are so ubiquitous and virulent in this environment [36, 37] that the risk of contracting a communicable infection for children is greatest immediately after entering a new group care arrangement [27, 38]. The fall in the incidence of such infections later in the year may indicate that the children have acquired some immunity to these pathogens or that the microbes in question are more prevalent in the summer months. There was, however, no significant increase in mucosal immunoglobulin levels observed in association with the increase in episodes of GIT symptoms. The immune system of the young child is in a constant stage of development and maturation and therefore is most vulnerable to environmental exposure. Microbial exposure early in life may potentially influence the development of immune networks in the airways [39], or the type of GIT flora encountered may modulate mucosal immune responses [40]. Such events at mucosal surfaces can impact not only on local responses, but also on responses at distant sites [2]. Central to the “hygiene hypothesis” [41] is the concept that overt infections and exposure to nonviable microbial compounds in the environment might confer protection from the development of allergic illness. In population based studies, various markers of infection burden, such as daycare attendance in infancy [42–46], infection, and the number of older siblings [44, 46–49], have been shown to be inversely associated with atopy-related disorders. The total burden of recurrent RT infections during the early years of life have also been shown to be negatively associated with atopy at pre-school [45] and at school age, in cross-sectional surveys [47, 49]. Among children at risk for atopic-related illness, exposure to rhinitis, croup, and recurrent ear infection early in life shows protection from atopy at school age [50–52]. Whilst a large number of epidemiological and longitudinal studies have looked at the “hygiene hypothesis” and the development of allergic illnesses, the question as to whether exposure to early infections has a causal or protective effect for future allergic disease remains controversial [22, 53]. However, as a result of these ongoing investigations, this hypothesis has evolved in various ways to show that the relationship between early-life illnesses and allergic disease is complex with emerging concepts related to the underlying innate and adaptive immune response mechanisms involved. In this study group, it was found that atopic disease was strongly associated with low levels of salivary IgA and IgG. In previous studies from our group, we have demonstrated that bronchial hyperreactivity was associated with the extent of salivary IgA deficiency in the first year of life [15] and that children born to atopic parents have significantly lower levels of salivary IgA compared with children of non-atopic parents [54]. The incidence of allergic disease in this study was similar to that which we observed in our earlier studies [54]. There is now evidence to suggest that mucosal IgA may have a protective role against the development of allergic sensitization in young children [55, 56], although, consensus is yet to be reached [33]. Children who had previous exposure to daycare were significantly more likely to have higher salivary IgA and IgM levels. The increase in salivary IgA reflects gut stimulation and the repertoire of gut-associated antigens [57], while salivary IgM [9, 18, 32, 58] has been occasionally observed in infants and adults in a pattern consistent with the concept that the occurrence of IgM in mucosal secretions reflects immune responses to novel antigens presented at mucosal sites, particularly the GIT [59]. Interestingly, reduced levels of salivary IgG were associated with increased pre-school exposure. One possible explanation is that the mucosal immune response is downregulated in response to high levels of antigen exposure [60]. Of the children in the current study that had a medical history of being hospitalised, over half of the hospitalisations were a result of infections or complications related to infections during the first two years of life (data not shown). The low salivary IgM observed in this subgroup may identify a risk factor for susceptibility to infection in these children, and further studies are required to determine possible mechanisms causing these lower levels of mucosal IgM in young children prone to infection. In another study [9] where children were hospitalised predominantly for surgical procedures, the IgM antibody levels were similar to those observed in nonhospitalised children. Higher levels of *E. coli-*specific IgA antibodies were strongly associated with children who had previous daycare experience and attended pre-school for two or more days a week compared to those that attended less frequently. This corresponds with the previous finding that, for pre-school-aged children, the number of hours spent in group care settings increases the likelihood of a child contracting a GIT illness [28], most probably due to increased contagion exposure. Interestingly, there was a sex-related difference in the immune response with respect to girls having lower levels of IgA specific antibody to *E. coli.* This suggests that the girls may have been exposed to lower levels of GIT pathogens and corresponds to the findings that boys have a higher incidence of contracting a GIT illness [38, 61]. Surprisingly, there was no association found between the number of siblings in the family and salivary immunoglobulin or *E. coli*-specific IgA antibody levels and suggests that close social interaction outside the home is a greater determinant of mucosal immune stimulation.

Perhaps the most ubiquitous and hazardous of children's environmental risks is exposure to ETS. Extensive literature links passive smoking with a wide range of effects on mortality and morbidity in children [62–65]. Exposure to cigarette smoke has been associated with abnormal mucosal development in children [66]; mucosal immune dysregulation in infants [67]; airway irritation that can result in local epithelial damage [68, 69]; airway inflammation which can adversely affect mucocilliary clearance [69, 70]; increased risk of carriage of potentially pathogenic bacteria [71] and dental caries [72] in children. Higher mucosal IgA levels in the infants of smokers have been attributed to more chronic upper RT symptoms [32, 33] which may be associated with increased adherence of respiratory pathogens to buccal epithelial cells [73]. In our study cohort, exposure to ETS was associated with elevated salivary IgA and IgG concentrations. In contrast to our observations, another study [74] found that the salivary IgA concentration was decreased in 4–6-year-olds exposed to ETS. The apparent disparity in results may be due to the use of different saliva collection and assay methods, or more importantly, the cohort of children studied. In the study by Avşar et al. [74], children who attended kindergarten were excluded from the study, as a consequence, these children in their study may not have experienced the same degree of antigenic challenge as the children in our study.

In conclusion, this study clearly demonstrates that exposure of children to environmental stimuli and noxious agents such as tobacco smoke has a profound effect on mucosal immune status and the potential for clinical sequelae. A large birth cohort study is currently being conducted in which the ontogeny profiles of salivary IgA and specific salivary IgA antibodies to common respiratory pathogens from birth to 5 years of age are being determined. This study will examine the effect of upper airway microbial colonization, ETS exposure, and other environmental factors on mucosal immune status and susceptibility to otitis media. A clear understanding of the way in which these factors interact with the mucosal system to influence development and control of immune function will assist in developing disease management strategies.

##  Disclosure

There is no conflict of interests to declare.

## Figures and Tables

**Table 1:** Demographic characteristics of the study population (*n* = 37).

Variables	Definition and description	Distribution
*Demographic variables*		

Age	≤4 years of age	15 (45.5%)
	>4 years of age	22 (54.5%)
Gender	Female	21 (56.8%)
	Male	16 (43.2%)
Having attended childcare previously	No	8 (21.6%)
	Yes	29 (78.4%)
No. of days at pre-school	Low—<2 days/week for <1 year	13 (35.1%)
	High—≥2 days/week for ≥1 year	24 (64.9%)
*Household environment variables*		

Having siblings	No	16 (43.3%)
No. of siblings	Yes	21 (56.7%)
		2.51^a^ (0.77)
ETS exposure in home	No	23 (63.2%)
	Yes	14 (37.8%)
*Medical history*		

Having atopy	No	31 (83.8%)
	Yes	6 (16.2%)
Having been hospitalised	No	23 (62.2%)
	Yes	14 (37.8%)

^a^The arithmetic mean of the number of siblings (and standard deviation) is reported for this covariate.

**Table 2:** Salivary immunological parameters, reported as geometric mean and 95% confidence interval (CI), at each assessment point during the pre-school year.

	Saliva collection time				

	1	2	3	4	5
	February (summer end) geometric mean (95% CI)	April (autumn) geometric mean (95% CI)	July (winter) geometric mean (95% CI)	September (spring) geometric mean (95% CI)	December (presummer) geometric mean (95% CI)
IgA (mg/L)	21.83 (18.99–25.10)	21.07 (18.20–24.40)	30.27 (25.72–35.61)	20.65 (17.48–24.39)	22.12 (18.85–25.97)
IgG (mg/L)	2.99 (2.34–3.82)	2.89 (2.29–3.66)	2.32 (1.86–2.91)	3.03 (2.26–4.06)	2.87 (2.16–3.82)
IgM (mg/L)	0.87 (0.64–1.17)	0.96 (0.73–1.26)	0.81 (0.63–1.04)	1.04 (0.83–1.31)	1.10 (0.81–1.48)
*E. coli*-specific IgA antibody (EU/mL)	0.11 (0.09–0.14)	0.08 (0.06–0.09)	0.10 (0.09–0.12)	0.12 (0.09–0.16)	0.11 (0.09–0.13)

**Table 3:** Regression coefficients (standard errors in parentheses) for multilevel regressions on each immunological outcome.

	Immunological outcome			
Predictor	IgA	IgG	IgM	*E*. *coli *
Time effect linear	0.172* (0.071)	−0.103 (0.109)	−0.021 (0.121)	−0.058 (0.099)
Time effect curvilinear	−0.043* (0.017)	0.021 (0.026)	0.018 (0.029)	0.020 (0.024)
Aged older than 4 years	0.271*** (0.081)	0.152 (0.132)	0.3218 (0.139)	0.091 (0.119)
Female	−0.035 (0.089)	0.159 (0.147)	0.120 (0.155)	−0.279* (0.132)
Previous childcare experience	0.185* (0.092)	−0.040 (0.150)	0.473** (0.157)	0.329* (0.136)
High pre-school exposure	−0.166 (0.090)	−0.317* (0.146)	−0.211 (0.154)	0.401** (0.132)
Having siblings	0.028 (0.056)	−0.107 (0.092)	0.130 (0.097)	0.070 (0.084)
ETS exposure	0.205* (0.082)	0.453*** (0.134)	0.087 (0.141)	−0.075 (0.121)
Having atopy	−0.216* (0.103)	−0.372* (0.168)	−0.025 (0.177)	0.199 (0.151)
Having been hospitalised	−0.002 (0.096)	0.151 (0.157)	−0.412* (0.165)	−0.125 (0.141)

*Note*. Outcomes are logtransformed.

Significance of associations: *P < .05; **P < .01; ***P < .001.
